# Circadian Clock and Sirtuins in Diabetic Lung: A Mechanistic Perspective

**DOI:** 10.3389/fendo.2020.00173

**Published:** 2020-04-03

**Authors:** Shuang Zhou, Yi-Min Dai, Xiao-Feng Zeng, Hou-Zao Chen

**Affiliations:** ^1^Department of Rheumatology, National Clinical Research Center for Dermatologic and Immunologic Diseases (NCRC-DID), Peking Union Medical College Hospital, Chinese Academy of Medical Sciences and Peking Union Medical College, Beijing, China; ^2^State Key Laboratory of Medical Molecular Biology, Department of Biochemistry and Molecular Biology, Institute of Basic Medical Sciences, Chinese Academy of Medical Sciences and Peking Union Medical College, Beijing, China

**Keywords:** circadian clock, Sirtuins, diabetic lung, oxidative stress, inflammation, aging

## Abstract

Diabetes-induced tissue injuries in target organs such as the kidney, heart, eye, liver, skin, and nervous system contribute significantly to the morbidity and mortality of diabetes. However, whether the lung should be considered a diabetic target organ has been discussed for decades. Accumulating evidence shows that both pulmonary histological changes and functional abnormalities have been observed in diabetic patients, suggesting that the lung is a diabetic target organ. Mechanisms underlying diabetic lung are unclear, however, oxidative stress, systemic inflammation, and premature aging convincingly contribute to them. Circadian system and Sirtuins have been well-documented to play important roles in above mechanisms. Circadian rhythms are intrinsic mammalian biological oscillations with a period of near 24 h driven by the circadian clock system. This system plays an important role in the regulation of energy metabolism, oxidative stress, inflammation, cellular proliferation and senescence, thus impacting metabolism-related diseases, chronic airway diseases and cancers. Sirtuins, a family of adenine dinucleotide (NAD^+^)-dependent histone deacetylases, have been demonstrated to regulate a series of physiological processes and affect diseases such as obesity, insulin resistance, type 2 diabetes (T2DM), heart disease, cancer, and aging. In this review, we summarize recent advances in the understanding of the roles of the circadian clock and Sirtuins in regulating cellular processes and highlight the potential interactions of the circadian clock and Sirtuins in the context of diabetic lung.

## Introduction

Diabetes mellitus appears to be one of the most common chronic diseases worldwide and leads to high premature mortality in human. The complications of diabetes can be separated into two main subgroups, microvascular complications, and macrovascular complications. Vascular damage plays a central role in diabetic complications. Despite a large capillary network, the lung is frequently overlooked because of its subclinical characteristic in diabetic patients. Recently, accumulating evidence has indicated a correlation between diabetes and impaired pulmonary structures and functions. In addition, diabetes increases the risk of some chronic pulmonary diseases. For instance, T2DM can deteriorate the progression and prognosis of COPD (1–4). Moreover, diabetes increases the severity of pulmonary hypertension secondary to COPD (5). A multicenter investigation proved that diabetes increases the odds of mortality for COPD patients (3). Therefore, the lung is certainly a diabetic target organ.

Mechanisms of lung damage caused by diabetes are still unclear, but some are convincing. For instance, glycosylation of proteins in the lung and chest wall promotes collagen accumulation in lung connective tissue and ultimately leads to a reduction in lung compliance (6–8). Hyperglycemia triggers vascular oxidative damage resulting in a loss of microvascular reserve in the lung. Systemic inflammation exaggerates vascular damage through endothelial dysfunction (9–11). Insulin resistance has been shown to disturb lung volume through leptin (12, 13). Interestingly, the anatomical and biological changes in the diabetic lung are similar to those described in the aging lung (14, 15), suggesting that mechanisms associated with premature aging may contribute to diabetic pulmonary injuries.

Mammalian Sirtuins, a family of adenine dinucleotide (NAD^+^)-dependent histone deacetylases, play important roles in age-related diseases including T2DM (16, 17). According to previous studies, Sirtuins are speculated to act on all of the known mechanisms underlying diabetic pulmonary injuries. However, few articles refer to the roles of Sirtuins in pulmonary pathophysiology, let alone in diabetic pulmonary injuries.

The circadian clock system drives mammalian intrinsic biological oscillations with a period of near 24 h (18). Current studies highlight the critical role of the circadian clock system in regulating cellular processes such as metabolism, oxidative stress, inflammation, cellular proliferation, and senescence (19, 20). Disrupted circadian rhythms are common in patients with chronic airway diseases and may trigger cellular senescence, especially among tobacco smokers, and disturb inflammatory responses in the lungs of COPD patients (21). Sirtuins regulate both the circadian clock in the brain as well as in peripheral tissues, including the lungs. Thus, it is rational to hypothesize that Sirtuins affect diabetic pulmonary injuries by interacting with the circadian clock system.

In this review, we provide a general view of the regulatory effects of Sirtuins and the circadian clock system in pulmonary pathophysiology and diabetic pulmonary injuries. Moreover, we focus on the interactions of Sirtuins and the circadian clock system to provide new ideas for viewing diabetes complications in the lung and to provide novel targets for therapies.

## The Lung Is a Diabetic Target Organ

### Histological Changes in the Diabetic Lung

Microangiopathy is a well-known diabetic complication involving the retina, kidney and peripheral or autonomic nervous system. A study comparing the histological changes in the lungs of diabetic patients showed significantly increased thickness of alveolar epithelial basal lamina (BL), endothelial capillary BL, and both fused BL (22). Moreover, researchers found the same thickening magnitude of BL in the lung and kidney in this study. The clinical findings of diabetic pulmonary microangiopathy have also been demonstrated in a streptozotocin (STZ)-induced diabetic rat model (23). In addition to microangiopathy, the increased glycosylation of insoluble collagen in human lung parenchyma found in young diabetic patients is similar to that in non-diabetic aged individuals (24). This phenomenon has also been reported in STZ-induced diabetic rats (25). These findings show accelerated aging in the diabetic lung. Data from a retrospective longitudinal cohort study shows that lung fibrosis is significantly enhanced in diabetic subjects (26). Lung fibrosis is representative of an important cause of premature mortality in patients.

### Function Abnormalities in the Diabetic Lung

Lung function mainly consists of pulmonary diffusion function and pulmonary mechanical function. Pulmonary diffusing capacity for carbon monoxide (DLCO) was reported to be reduced among diabetes patients compared with healthy subjects (27). The reduction in DLCO in diabetic patients is parallel to the severity of retinopathy and nephropathy (15). Moreover, glycemic control increases DLCO (28). Pulmonary mechanical function is reflected by several parameters, including forced expiratory volume in 1 s (FEV1), forced vital capacity (FVC), and total lung capacity (TLC). An increasing number of recent studies support the notion that there is a correlation between diabetes and decreased pulmonary mechanical function (28–30). The meta-analysis carried by Klein et al., showed a decline in lung function, including FEV1, FVC, and DLCO, in diabetic patients compared with healthy subjects (31). The reduction in lung function is negatively correlated with blood glucose level and the duration and severity of diabetes (31). In four longitudinal studies, two studies demonstrated a significant decline in lung function in diabetic patients compared with healthy individuals, and two other studies showed lower FEV1 and FVC in patients before diabetes onset than in subjects who did not develop diabetes (31).

**Figure 1:**
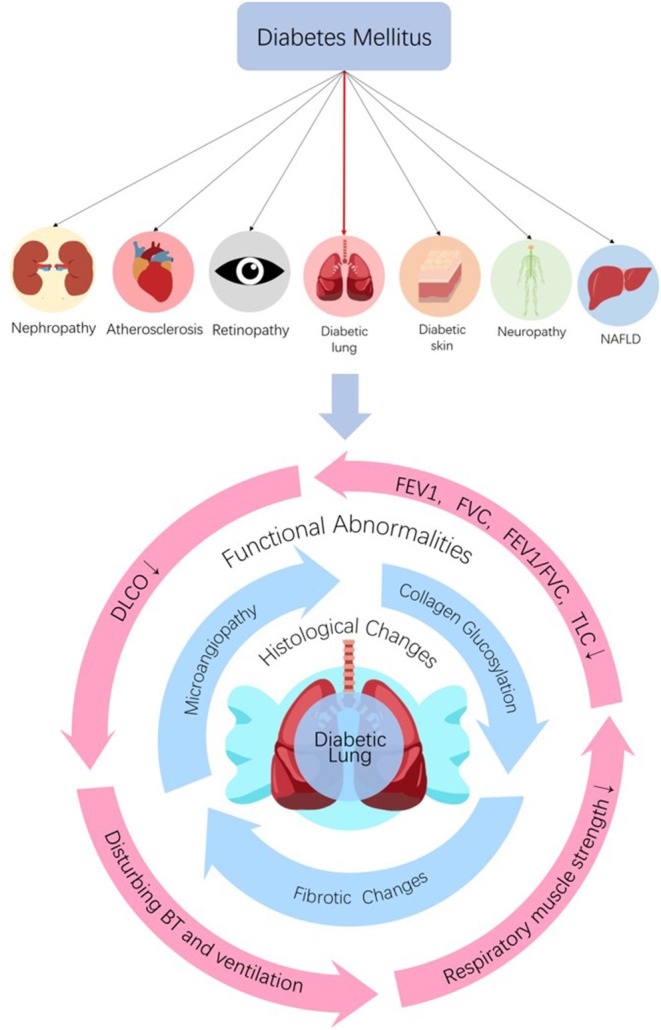
The lung is a diabetic target organ. Diabetes can induce tissue injuries in target organs including nephropathy, retinopathy, neuropathy, NAFLD, dermopathy, and atherosclerosis in coronary arteries, cerebrovascular and peripheral vascular systems. Despite the subclinical characteristic of the lung, diabetes induces pulmonary structural and functional abnormities, so the lung is a diabetic target organ. Diabetes can induce histological and functional changes to the lung. Histological changes, such as microangiopathy, fibrotic changes, and accelerated collagen aging, can be seen in diabetic lungs. Impaired pulmonary functions such as reductions in FEV1, FVC, FEV1/FVC, TLC, and DLCO, abnormal bronchomotor tone, decreased ventilatory response to hypercapnia and reduction in the strength of respiratory muscle have been implicated in the diabetic lung. NAFLD, non-alcoholic fatty liver disease; FEV1, forced expiratory volume in 1 s; FVC, forced vital capacity; TLC, total lung capacity; DLCO, diffusing capacity for carbon monoxide; BT, bronchomotor tone.

The bronchomotor tone (15, 32–34), the chemosensibility to hypoxia (35, 36) and the respiratory muscle strength (37–40) are damaged in diabetic patients. On the basis of histological and functional changes in the lungs of diabetic patients and animals, it can be concluded that the lung is a definite diabetic target organ (Figure 1).

## Molecular Mechanisms Underlying Diabetic Pulmonary Injuries

**Figure 2:**
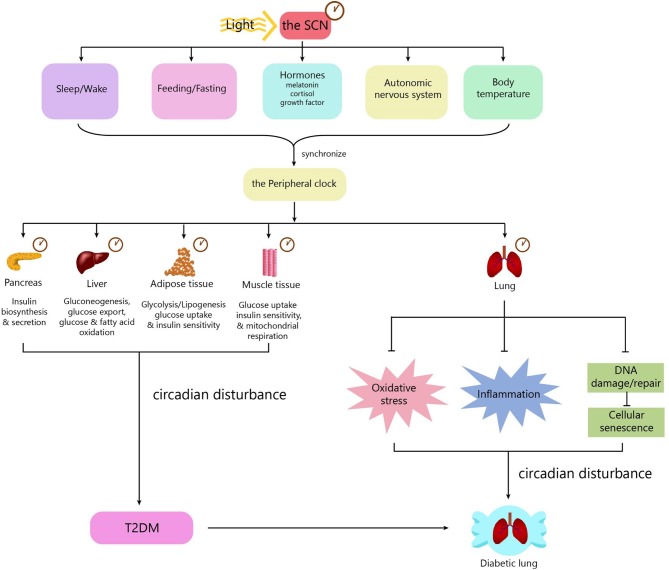
The role of the circadian clock in the diabetic lung. The circadian clock system consists of a central clock located in the SCN and peripheral clocks located in the pancreas, muscle, liver, adipose tissue, gut, kidney, and lung. Light is the most important zeitgeber for the SCN. The SCN synchronizes the peripheral clocks through signals including biological behaviors, hormones, the autonomic nervous system, and body temperature. The central clock and peripheral clocks regulate insulin secretion, insulin sensitivity, lipid, and glucose metabolism. Circadian disturbance contributes to the development of T2DM. The peripheral clock in the lung can influence some pathophysiological processes, including oxidative stress, inflammation, and cellular senescence. Disturbance of the circadian clock in the lung promotes the development of diabetic lung. SCN, suprachiasmatic nucleus; T2DM, type 2 diabetes.

Mechanisms underlying diabetic pulmonary injuries remain unclear; however, advanced glycosylation end-products (AGEs), oxidative stress and inflammation, endothelial dysfunction, and hypercoagulation convincingly contribute to pulmonary injuries, which have well-summarized in other reviews (15, 41–43). All of the above-mentioned pathogenic factors are regulated in a circadian manner. Patients with chronic airway diseases, including COPD and asthma, develop more frequently, and worsen mostly in the evening or early morning (44–46). Accumulating evidence shows that circadian rhythm regulation is upstream of known mechanisms. Next, we will discuss the mechanisms underlying diabetic pulmonary injuries in the context of circadian regulation (Figure 2).

## Circadian Regulation in the Diabetic Lung

### The Circadian Clock System

In mammals, the circadian clock system consists of a central clock located in the hypothalamic suprachiasmatic nucleus (SCN) and peripheral clocks, which drive 24 h rhythms of physiology and behavior. The SCN clock is set mainly by environmental light and then sends the entrained timing signal to peripheral clocks via neural signals, hormonal signals, and body temperature. At the cellular level, the circadian rhythms are generated by clock genes. The core clock genes include *Bmal1* and *Clock* encoding activators, period genes (*Per1-3*) and cryptochrome genes (*Cry1-2*) encoding repressors and genes encoding the nuclear receptors REV-ERB (*NR1D1* and *NR1D2*) and ROR (*Ror*α, *Ror*β, and *Ror*γ).

Clock genes forms a transcriptional autoregulatory feedback loop. The BMAL1/CLOCK heterodimer translocate to the nucleus and transcriptionally activate expression of the core clock genes including *Per1-3, Cry1-2*, and nuclear receptors *Rev-erb*α and *Ror*α. Conversely, once PER and CRY accumulate to a certain level, they form heterodimer and translocate back to the nucleus to block transcriptional activity of the BMAL1/CLOCK complex and ultimately repress their own transcription. REV-ERB and ROR drive the rhythmic expression of BMAL1 and CLOCK via competitively binding to the REV-ERB/ROR binding site, thus repressing or activating transcription of *Bmal1* and *Clock*, respectively (47, 48). In addition, posttranslational modifications have been established to regulate clock gene expression. For instance, SIRT1 binds BMAL1/CLOCK and promotes deacetylation and degradation of PER2 (49). Phosphorylation of PER2, mediated by casein kinase Iε, recruits the ubiquitin ligase adaptor protein β-TrCP and leads to polyubiquitination and proteasome-mediated degradation of PER2 (50). CRY binds PER2 and prevents its nuclear export, thus preventing the ubiquitylation and subsequent degradation of PER2 (51). Likewise, PER2 can prevent the ubiquitylation and subsequent degradation of CRY (51). CLOCK can induce sumoylation of BMAL1 at Lys259 and control BMAL1 stability (52). Both BMAL1 and CLOCK undergo phosphorylation during the circadian cycle (53), which is coupled to nuclear translocation and the subsequent degradation of CLOCK (54). CLOCK has intrinsic histone acetyltransferase activity and can acetylate BMAL1 on the Lys537 residue, which facilitates the recruitment of CRY1 to the BMAL1/CLOCK complex, resulting in transcription repression (55).

These core clock genes function not only as active or repressive components of a cell-autonomous clock but also as regulators of clock-controlled genes (CCGs). Mechanistically, the core clock genes interact with chromatin-modifying complexes, co-activators and co-repressors to regulate CCG expression. The BMAL1/CLOCK complex drives the expression of numerous CCGs, thus regulating a series of biological processes, including metabolism. At the beginning of transcription, the BMAL1/CLOCK complex interacts with chromatin and recruits chromatin-modifying complexes such as histone acetyltransferases P300 and CBP (56), histone deacetylases SIRT1 (57, 58) and SIRT6 (59), methyltransferases MLL1 (60) and MLL3 (61), and histone lysine demethylases JARID1a and LSD1 (62) to promote chromatin accessibility and activate CCG transcription. The PER-CRY repressor complex translocate to the nucleus and recruits a series of co-repressors to block BMAL1/CLOCK complex activity (48). In general, in the circadian cycle, transcriptional activation and repression of rhythmic genes involve dynamic chromatin epigenetic transition. In addition, transcription factors, including NF-κB, nuclear receptor hepatocyte nuclear factor 4A (HNF4A) and USF1, can compete with the BMAL1/CLOCK complex for binding to target genes and repress the transcriptional activity of the BMAL1/CLOCK complex (63–65). MYC can inhibit the expression and oscillation of BMAL1 by inducing REV-ERBα expression (66). Furthermore, REV-ERBα can recruit the N-CoR/HDAC3 co-repressor to regulate the expression of some metabolic genes (67). Conversely, transcription factors, including PDX1 and HIF1α, act synergistically with BMAL1 to activate target gene expression (68, 69).

### The Role of the Circadian System in Diabetes

As described in a series of reviews, the circadian clock system plays a pivotal role in regulating energy metabolism and maintaining energy homeostasis. The SCN clock drives sleep-wake and feeding-fasting cycles and functions as the basic biological clock of metabolism. Except for tuning by the SCN, peripheral clocks can be set by feeding as well, having autonomic circadian oscillators in their respective tissues and contributing to metabolic processes. Insulin resistance and pancreatic β cell dysfunction are critical pathophysiological processes in the development of T2DM. Glucose metabolism and insulin secretion occur in a circadian manner. Internal circadian system dysfunction induces insulin resistance and glucose intolerance. The roles of the SCN clock and peripheral clocks located in different tissues in insulin secretion, insulin sensitivity and glucose metabolism regulation will be discussed individually.

First, the SCN clock controls the sleep-wake cycle as well as rhythmic feeding behavior, which is critical in determining organism nutritional status and in the development of diabetes. The SCN dives the rhythmic release of several hormones that affect the secretion and/or action of insulin. For instance, melatonin, a hormone synthesized by the pineal gland at night, is orchestrated by output from the SCN and coordinates circadian activity in turn by regulating clock gene expression (70). Furthermore, melatonin exerts its function through two specific receptors, MT1 and MT2, in different peripheral tissues. Both of these receptors are present in human islets. The protective roles of melatonin in maintaining glucose homeostasis and suppressing insulin resistance and T2DM have been described in substantial human studies. For instance, lower nocturnal melatonin secretion is linked with increased insulin resistance in non-diabetic individuals and is an independent risk factor for developing T2DM (71). Notably, diabetic patients mostly lack circadian melatonin rhythm (72). Specific single nucleotide polymorphisms of MT2 are related to higher fasting glucose levels and HbA1c (73, 74). Further, loss-of-function mutations of MT2 are associated with the highest incidence of T2DM (75). In addition to regulating insulin secretion, melatonin has other functions, such as stimulating antioxidant enzymes (76) and attenuating the production of proinflammatory cytokines in high-fat diet (HFD)-induced insulin-resistant rats (77), suppressing mitochondrial dysfunction in diabetic rats (78), reducing cortisol secretion (75), and regulating glucose metabolism in adipocytes (79), skeletal muscle cells (80), and hepatocytes (81). In addition, the SCN affects the production and release of cortisol via regulating the activity of the hypothalamic-pituitary-adrenal axis (HPA) (82). Endogenous hypercortisolism can cause pancreatic β cell dysfunction and induce insulin resistance in the liver, adipose tissue and skeletal muscle (83). Disorder of circadian rhythm caused by obstructive sleep apnea causes HPA hyperactivity, contributing to insulin resistance (84). The SCN regulates the diurnal rhythm of growth hormone, which exerts anabolic effects and favors body composition and physical fitness (85). Moreover, the SCN is responsible for circadian regulation of energy expenditure for thermogenesis (82).

Convincingly, pancreatic β cell dysfunction contributes to T2DM. As mentioned above, insulin from rodents (86) and human (87, 88) islet cells is secreted in a circadian manner. Insulin secretion lacking a rhythmic release pattern has been observed in T2DM patients (89). The pancreatic clock is synchronized to the light-dark cycle by the SCN via signals such as melatonin, cortisol and body temperature (86–88). Pancreatic islet cells in mice have self-sustained clock genes and protein oscillations of BMAL1 and CLOCK, which act with co-activator PDX1 to activate the transcription of genes involved in insulin biosynthesis, transport and secretion (68). Moreover, specific ablation of these clock components disrupted insulin secretion leading to diabetes in mice (90). Saini et al. reported that circadian clock disruption via small interfering RNA perturbed insulin secretion in human pancreatic islet cells (88).

Adipose tissue, liver and skeletal muscle are important insulin target organs responsible for energy metabolism, and insulin resistance in these organs contributes to the development of T2DM. These organs have autonomous circadian clocks that are synchronized by the SCN (91) and signals from food intake (92–96). Misalignment of the peripheral clocks in these organs by disruption of the normal fasting-feeding cycle contributes to the development of diabetes in HFD-fed mice (97). Furthermore, germ-line *Bmal1* disruption mice exhibit increased total fat content, glucose intolerance comparable to mice lacking protein kinase Akt2 (98) as well as reduced insulin production after refeeding following overnight fasting (99). Adipocytes from humans present rhythmic glucose uptake due to an intrinsic diurnal rhythm in insulin sensitivity (96), which has been mechanistically demonstrated to be associated with circadian regulation of retinol-binding protein receptor STRA6 (100). The circadian clock regulates the expression of key enzymes involved in lipolysis (101) and lipogenesis (102), and disruption of the clock promotes triglyceride accumulation in white adipose tissue (101). As the liver plays a pivotal role in maintaining blood glucose homeostasis by regulating glycogenolysis, glycogenesis, and gluconeogenesis, it is strongly affected by the fasting-feeding cycle. Abundant genes in the liver responsible for glucose metabolism exhibit circadian regulation (103). Liver-specific *Bmal1* knockout (KO) mice showed abnormalities in both glucose storage and production resulting from disturbed expression of CCGs, including *Glut2, GCK, Pepck2*, and *L-PK*, which confirms the essential role of the liver clock in maintaining euglycemia (99). Human muscle exhibits diurnal rhythms in mitochondrial oxidative capacity and insulin sensitivity (104, 105). Muscle-specific *Bmal1* KO mice showed insulin resistance and impaired glucose metabolism in skeletal muscles (106). The BMAL1/CLOCK complex regulates the expression and membrane translocation of the insulin-sensitive glucose transporter GLUT4 and affects pyruvate dehydrogenase (PDH) activity by regulating the expression of PDH regulators, including *Pdp1* and *Pdk4*, ultimately impacting glucose oxidation (106). In addition, the BMAL1/CLOCK complex improves insulin sensitivity through the upregulation of SIRT1 expression in cultured C2C12 myotubes and mouse skeletal muscle (107). Recently, the muscle clock was reported to regulate insulin sensitivity and glucose utilization by affecting genomic recruitment of HDAC3 and subsequently disturbing the expression of metabolic genes (108).

### The Role of the Circadian System in the Lung

As early as two decades ago, researchers observed clock gene expression in the lungs of rats (109). Later, the link between circadian rhythm and lung pathophysiology was well-documented. For example, patients with asthma show a circadian rhythm in the bronchial response to challenges such as cold dry air, dust mite, histamine, etc. (110). Nocturnal worsening in lung function in asthma has been linked to diurnal alterations of inflammation and airway narrowing (111). Core clock genes are expressed strongly in Clara cells lining the bronchioles, and these cells are critical for maintaining circadian oscillations in both mouse and human lung tissue (112). Subsequent studies declared that environmental factors such as air pollutants, cigarette smoke (CS), allergens, pathogens, jet lag, and shift work can disturb molecular clock function in the lung and lead to exacerbations of chronic lung diseases, including COPD, lung fibrosis and asthma (113–120).

#### The Role of the Circadian Clock in Pulmonary Redox Regulation

A growing body of evidence indicates that the molecular clock regulates redox in multiple tissues. For instance, global *Bmal1* KO mice showed significant ROS accumulation in the kidney, heart, brain, and spleen compared to wild-type mice, indicating that BMAL1 controls ROS homeostasis (121). These *Bmal1*^−/−^ mice showed reduced expression of redox genes, including *Aldh2* encoding ALDH2, which scavenges reactive aldehydes generated during mitochondrial respiration, and *Nqo1* encodes NADPH dehydrogenase, which decreases toxic quinones and ultimately increases lipid peroxidation in the brain, promoting neurodegeneration (122). *Bmal1* depletion in macrophages reduced the NRF2 response to LPS challenge, resulting in ROS accumulation and production of the proinflammatory factors IL-1β and IL-6 (123). Furthermore, *Bmal1* depletion predisposes pancreatic β cells to oxidative-induced β cell dysfunction, generating a diabetic phenotype in mice (124). In humans, impaired redox balance has been associated with several chronic pulmonary diseases, including COPD, lung fibrosis, asthma, and lung cancer (125–128), which contributes to diabetic pulmonary dysfunction, as previously mentioned. The critical role of NRF2 in cellular antioxidant defense has been well-documented in a substantial number of studies. In response to oxidative stress, NRF2 translocates to the nucleus and induces the expression of a series of antioxidant genes, including glutathione cysteine ligase (GCL), glutathione S-transferase (GST), and haeme oxygenase 1 (HMOX1) (129, 130). In ClockΔ19 mice expressing a dominant negative mutation of the CLOCK protein, NRF2 expression in the lung is constitutively low and arrhythmic and is accompanied by reduced glutathione levels, increased markers of oxidative damage and fibrotic phenotype (131). Several antioxidant enzymes, including SODs and GPxs, are under transcriptional control of PPARs (132–137). Interestingly, PPARα is involved in a positive regulatory feedback loop with BMAL1 in rodent liver circadian clock (138). PER2 interacts with PPARγ directly and represses its activity (139). These findings suggest that the circadian clock may exert an antioxidant role by regulating PPARs. In addition, the circadian clock has been shown to drive NAD^+^ oscillations and control mitochondrial oxidative metabolism (140). REV-ERBα is reported to improve skeletal muscle oxidative capacity by reducing mitochondrial autophagy and biogenesis (141). As mitochondrial dysfunction is the major cause of ROS production, the circadian clock is supposed to protect against oxidative damage to the diabetic lung via regulating mitochondrial function.

#### The Role of the Circadian Clock in the Pulmonary Inflammatory Response

The molecular clock powerfully regulates the inflammatory response (142, 143). Global *Bmal1* KO mice display significantly increased expression of proinflammatory cytokines, including TNF-α, COX2 and prostaglandin synthase gene (*ptgs2*), in the brain, suggesting that molecular clock disruption is directly related to the inflammatory response (122). Myeloid cell-specific *Bmal1* depletion disrupts rhythmic mobilization of Ly6Chi monocytes and fortifies inflammatory responses, potentiating metabolic inflammation, and predisposing experimental animals to insulin resistance and metabolic dysfunction (144). Mechanistically, the BMAL1/CLOCK complex recruits polycomb repressive complex 2 (PRC2) to chemokine gene promoters, such as *Ccl2, Ccl8*, and *S100a8*, and silences expression of these CCGs in monocytes and macrophages (144). The BMAL1/CLOCK complex induces NRF2 expression via binding the E-box sites in the *Nrf2* promoter, which contributes to suppressing the inflammatory response of macrophages (123). NRF2 further inhibits proinflammatory cytokine IL-6 and IL-1β expression by reducing ROS levels (123) as well as by inhibiting the recruitment of RNA polymerase II to the transcription start sites (TSSs) of *IL-6* and *IL-1*β (145). BMAL1 can exert an anti-inflammatory effect by recruiting glucocorticoid receptors to promoters of proinflammatory cytokines such as CXCL5 (116). Bronchiole-specific *Bmal1* depletion enhances CXCL5 expression, driving pulmonary neutrophil recruitment and augmenting pulmonary inflammation and responses to pathogen (116). Deletion of *Bmal1* in pulmonary airway epithelial cells increases neutrophil infiltration in mouse lungs, alters lung mechanic functions and impairs influenza defense (146). An HFD has been shown to induce insulin resistance partly via activation of the NF-κB signaling pathway (147). CLOCK interacts with the p65 subunit of NF-κB and enhances NF-κB-dependent transcription; however, BMAL1 counteracts NF-κB activation by sequestering CLOCK (148). Positively regulated by BMAL1, the nuclear receptor REV-ERBα has been demonstrated to be an important intermediary molecule linking the core clock and inflammatory pathways in macrophages. As a transcriptional repressor, REV-ERBα inhibits IL-6 and Ccl2 production by direct DNA binding to their promoters (149, 150). REV-ERBs repress macrophage genes, including *Mmp9* and *Cx3cr1* expression, by inhibiting the function of distal enhancers selected by macrophage lineage-determining factors (151). Deletion of *Rev-erb*α in bronchial epithelia exaggerates pulmonary inflammation (152). Inflammatory stimuli can induce REV-ERBα protein degradation, which can be blocked by its inverse agonist GSK1362 (152). Deletion of *Cry* releases its inhibition of cAMP production and leads to constitutive activation of PKA, which results in activation of NF-κB via phosphorylation of the p65 subunit and subsequent induction of proinflammatory cytokines (153).

In recent years, Although the role of the circadian clock has been well-documented in the development of insulin resistance and T2DM via the regulation of oxidative stress, inflammation and energy metabolism, far less is known about circadian regulation in the diabetic lung. SIRT1, a well-known member of Sirtuins, has been well-demonstrated to bridge metabolism and circadian rhythms, and together with other family members, SIRT1 plays a powerful role in metabolic homeostasis, oxidative stress, inflammation, and aging. Next, we will retrospectively examine the roles of Sirtuins in diabetic pulmonary injuries and discuss the interactions of Sirtuins with circadian clocks in this context.

## The Circadian Roles Of Sirtuins in the Lung

**Figure 3:**
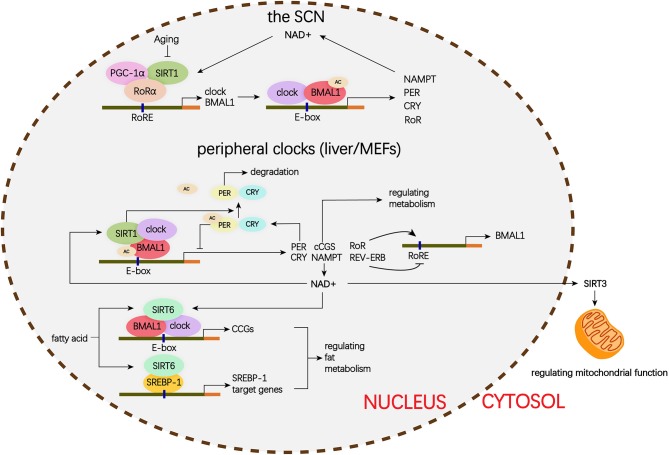
Schematic representation of the role of Siruins in clock regulation. Clock genes forms a transcriptional autoregulatory feedback loop. The BMAL1/CLOCK heterodimer transcriptionally activate expression of the core clock genes including *Per1-3, Cry1-2*, and nuclear receptors *Rev-erb*α and *Ror*α. Conversely, once PER and CRY accumulate to a certain level, they form complex with BMAL1/CLOCK and block transcriptional activity of the BMAL1/CLOCK complex and ultimately repress their own transcription. In addition, REV-ERB represses and ROR activates *BMAL1* transcription. (1) In the SCN, the binding of SIRT1 and PGC-1α to the *Bmal1* promoter containing RORE through a synergistic action with RORα amplifies circadian gene expression, thereby, brain SIRT1 prevents circadian function decline with aging. (2) In the peripheral clock located in the liver and MEFs, there is a new negative feedback loop. BMAL1/CLOCK positively regulates the transcription of NAMPT, the rate-limiting enzyme in mammalian NAD^+^ biosynthesis. NAD^+^ is a classic co-enzyme for Sirtuins. Conversely, SIRT1 deacetylates BMAL1 and represses BMAL1/CLOCK activity. In addition, SIRT1 binds to BMAL1/CLOCK complex and promotes the deacetylation and degradation of PER2. SIRT6 controls different circadian genome subdomains from SIRT1. SIRT6 interacts with BMAL1/CLOCK complex and controls their chromatin recruitment to CCGS promoter. Meanwhile, SIRT6 governs circadian chromatin recruitment of SREBP-1. The core clock regulates circadian oscillation of SIRT3 activity by driving changes in NAD^+^ levels. MEFs, mouse embryonic fibroblasts; PGC-1α, peroxisome proliferator-activated receptor gamma co-activator 1-alpha; BMAL1, brain, and muscle aryl hydrocarbon receptor nuclear translocator-like 1; CLOCK, circadian locomotor output cycles protein kaput; PER2, period 2; SREBP-1, sterol regulatory element-binding protein 1; CCGs, clock-controlled genes; mTOR, mechanistic target of rapamycin; IGF, insulin-like growth factor; NF-κB, nuclear factor κB; mtDNA, mitochondrial DNA; TGFβ1, transforming growth factor β1; NAD, nicotinamide adenine dinucleotide; NAMPT, nicotinamide phosphoribosyl transferase; FOXO3, forkhead protein box 3; E-box, enhancer box; RORα, retinoic acid receptor– related orphan receptor a; RORE, retinoic acid receptor–related orphan receptor response element.

**Figure 4:**
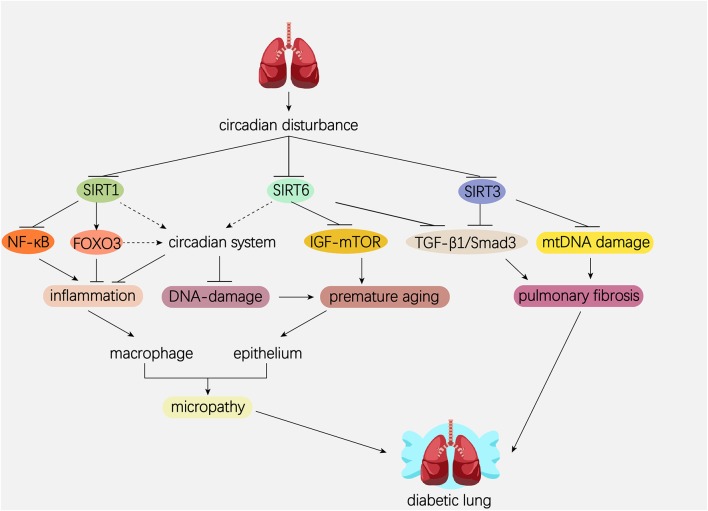
Hypothetical model showing the possible role of Sirtuins and circadian system in the pathogenesis of diabetic lung. Dotted line represents the pathway needed to be elucidated. Solid line represents the pathway which has been proved in former studies. Circadian disturbance decreases deacetylase activity of Sirtuins via impairing NAD^+^ oscillation. SIRT1 inhibits pulmonary inflammation through regulating circadian clock or through regulating transcriptional factors such as NF-κB and FOXO3. SIRT1 and SIRT6 rescue pulmonary cells from premature aging via regulating the DNA damage response synergistically with circadian clock. In addition, SIRT6 prevents premature aging by attenuating the IGF-mTOR signaling pathway. Both SIRT3 and SIRT6 inhibit pulmonary fibrosis by inactivating the TGFβ1/Smad3 signaling pathway. SIRT3 inhibits pulmonary fibrosis by maintaining mtDNA stability. NAD, nicotinamide adenine dinucleotide; NF-κB, nuclear factor κB; FOXO3, forkhead protein box 3; mTOR, mechanistic target of rapamycin; IGF, insulin-like growth factor; TGFβ1, transforming growth factor β1; mtDNA, mitochondrial DNA.

The mammalian Sirtuins are a family of NAD^+^-dependent deacetylases consisting of seven members (SIRT1-SIRT7) (154). These seven members display distinct subcellular localization and biological functions (17). Sirtuin family members regulate a wide variety of cellular processes and affect a series of diseases such as obesity, insulin resistance, T2DM, cardiovascular disease, cancer, and aging (155, 156). Sirtuins are supposed to prevent diabetic lung through attenuating diabetes. Roles of Sirtuins in insulin resistance, NAFLD and T2DM etc. have been summarized in our and other reviews (17, 155). Here, we will focus on the interaction of Sirtuins and the circadian clock (Figure 3) and discuss their possible role in diabetic pulmonary injuries (Figure 4).

### The Interaction of SIRT1 and the Circadian Clock in Diabetes and Pathogenesis of the Diabetic Lung

#### The Role of SIRT1 in the Central Clock

*Sirt1* mRNA is highly expressed in the hypothalamus (157), which is an important metabolism-relevant region as well as the location of the central clock. Loss of brain SIRT1 activity in mice results in an abnormal extension of the intrinsic period and an inability to reset a new light-dark regimen (158). Expression of core clock genes, including BMAL1, CLOCK, and PER2, in the SCN significantly decreases in brain-specific *Sirt1* knockout (BSKO) mice and increases in brain *Sirt1* transgenic mice (BSTG) (158). Mechanistically, in N2a neuroblastoma cells, SIRT1 and PGC-1α bind cooperatively to the *Bmal1* promoter, driving its expression and enhancing the amplitude of circadian machinery (158). In addition, SIRT1 in the ventromedial hypothalamus (VMH) has been shown to function as a metabolic sensor by sending nutritional information to the SCN via efferent signals and synchronizing the central clock to feeding conditions (159). *Sirt1* gene ablation in the Sf1 neurons of the VMH perturbs the activity and circadian gene expression in the SCN and subsequently disrupts the connection of food intake and circadian behavior (159). These studies support the crucial role of SIRT1 in linking metabolism with the central pacemaker.

#### The Role of SIRT1 in Peripheral Clocks

Studies on the interactions of SIRT1 and peripheral clocks have mainly been carried out in mouse liver cells and embryo fibroblasts (MEFs). First, SIRT1 is expressed in a circadian manner in mouse liver cells, MEFs, and NIH 3T3 cells and is essential for augmenting the expression of several core clock genes, such as *Bmal1, Ror*γ, *Per2*, and *Cry1* (49). This study also reported that SIRT1 binding to the BMAL1/CLOCK complex rhythmically drove the deacetylation and degradation of the PER2 protein (49). A secondary study revealed that SIRT1 deacetylase activity rather than transcript and protein levels was regulated in a circadian manner in mouse liver cells and MEFs (57). SIRT1 associates with CLOCK and is then recruited to the BMAL1/CLOCK complex at the CCG promoters, where it is responsible for the rhythmic deacetylation of H3 Lys9/Lys14 and BMAL1 at Lys537, counteracting the acetyltransferase activity of CLOCK (160), and prevents the transcriptional activating effect of the BMAL1/CLOCK complex (57). This study revealed the crucial role of SIRT1 in acting as the molecular rheostat of CLOCK. Subsequent studies declared that the SIRT1 activity rhythm was generated from the oscillation of intracellular NAD^+^ levels, which are regulated by NAMPT, the rate-limiting enzyme in the NAD^+^ salvage pathway (58, 161). NAMPT synthesis is positively regulated by the BMAL1/CLOCK complex, and SIRT1 activation due to NAMPT-mediated NAD^+^ biosynthesis, in turn, suppresses BMAL1/CLOCK complex activity, forming a circadian clock feedback loop (58, 161). In addition to histone acetylation, circadian transcription is associated with MLL1-mediated H3K4 trimethylation. MLL1-dependent H3K4me3 on the CCG promoter favors the recruitment of the BMAL1/CLOCK complex and activates CCG transcription (60). MLL1 exhibits rhythmic acetylation of two conserved lysine residues, K1130 and K1133, and SIRT1 deacetylates these two lysine residues of MLL1, inhibiting its methyltransferase activity (162). Some studies demonstrated other interactions of SIRT1 and clock genes. For instance, BMAL1/CLOCK regulates mouse hepatocyte insulin sensitivity via circadian regulation of the expression of SIRT1 (163). Similarly, BMAL1/CLOCK regulates muscle insulin sensitivity through circadian regulation of SIRT1 expression (107). PER2 negatively regulates mouse hepatocyte SIRT1 expression by binding to the BMAL1/CLOCK binding E-box sites in the *Sirt1* promoter *in vivo* and *in vitro*. In turn, *Sirt1* deficiency leads to increased acetylation of H4K16 in the *Per2* promoter and subsequent transcriptional activation of *Per2*, which results in misalignment of the circadian rhythm in the liver (164).

#### SIRT1 Regulates the Circadian Clock in Response to Inflammation in the Lung

SIRT1 protein levels and activity decrease in macrophages, lung epithelium and peripheral lung tissues of smokers and COPD patients, leading to increased acetylation of RelA/p65 and subsequent activation of the NF-κB pathway (165). SIRT1 activity in peripheral blood mononuclear cells is positively associated to lung function of COPD patients (166). Similar results were observed in the lungs of rats and mice exposed to CS (167, 168). BMAL1 levels also decrease in the lungs of COPD patients compared with non-smoking individuals (169). Acute and chronic CS exposure reduces the amplitude of core clock genes, especially BMAL1 (169). Moreover, lung epithelial-specific *Bmal1* deletion mice show enhanced pulmonary inflammation in response to CS (169). However, SRT1720, a pharmacological activator of SIRT1, failed to inhibit CS-induced pulmonary inflammation in lung epithelial-specific *Bmal1* deletion mice (169). These findings show a pivotal role of the SIRT1-BMAL1 pathway in regulating pulmonary inflammation in response to environmental stress (119, 169). Although SIRT1 may play an important role in regulating several pulmonary pathophysiological processes, including oxidative stress, inflammation, and endothelial dysfunction, which are involved in the development of the diabetic lung, studies about the direct effects of SIRT1 on the diabetic lung are absent.

#### Roles of SIRT1 and the Circadian Clock in Pulmonary Premature Aging

Interestingly, the anatomical and biological changes in the diabetic lung are similar to those described in the aging lung, which suggests that we can discuss the roles of SIRT1 and the circadian clock in the diabetic lung from the perspective of cellular senescence-induced organ dysfunction and aging.

CS is a well-known factor leading to rapid decline in lung function and increases cellular senescence in the lungs of COPD patients (167). Mechanistically, CS induces DNA damage and impairs double-strand break (DSB) repair (47, 170, 171). Persistent DNA damage in the lung causes stress-induced senescence (SIPS) and a senescence-associated secretory phenotype (SASP), as characterized in COPD (172, 173). As mentioned above, CS also induces molecular clock dysfunction in the lung. The molecular clock is established to play a critical role in regulating the cellular response to DNA damage (174, 175). Bmal1-deficient mice show age-related pathologies and increased levels of ROS in a series of organs (121). Circadian clock protein also mediates cellular DNA damage/repair responses by interacting with factors such as Ku70, Ku80 and ataxia telangiectasia mutated (ATM) (175–177). p21 is induced by p53 following DNA damage and is known as a prosenescence gene. The *p21* gene is negatively regulated by REV-ERBα, which in turn is positively regulated by the CLOCK/BMAL1 complex (178). Therefore, Bmal1 deficiency results in aberrant p21 expression and decreased hepatocyte proliferation (178). In addition, PER1 inhibits p21 expression and interacts with the checkpoint proteins ATM and Chk2, leading to significant growth reduction and sensitizing human cancer cells to DNA damage-induced apoptosis (179). These studies suggest that the circadian clock can influence cellular senescence by regulating the DNA damage response pathway.

In aged mice, levels of SIRT1 and core clock genes, including BMAL1, CLOCK, CYR1, and PER2, in the SCN decrease, leading to a longer intrinsic period and the inability to reset to a new light-dark regimen (158). Young BSKO mice have the same circadian changes as aged mice (158). SIRT1 and PGC-1α synergistically activate expression of the circadian activator *Bmal1* in the SCN (158). This study suggests that SIRT1 links the clock with aging in the mammalian brain. In response to DNA damage, SIRT1 is recruited to DSBs and is required for efficient DSB repair and genomic stability; however, this process results in deregulation of genes causing aging (167, 180, 181). SIRT1 overexpression promotes survival in a mouse model of genomic instability and inhibits age-related transcriptional changes (167, 180, 181). Furthermore, SIRT1 activation by both overexpression and pharmacological activator SRT1720 can reduce cellular senescence via the SIRT1-FOXO3 axis and then attenuate emphysema (167). This protective role of SIRT1 in emphysema is not attributed to its effect on NF-κB-mediated inflammation (167). CLOCK is reported to be a transcriptional target of FOXO3, and FOXO3 knockdown dampens circadian amplitude in the mouse liver (182). Hence, it is rational that SIRT1 affects SIPS and SASP by regulating molecular clock directly or indirectly in the lung. In addition, SIRT6 deletion also decreases genomic stability via reducing base excision DNA repair and causes accelerating aging in mice (183). Accumulating evidence indicates that SIRT1 and SIRT6 play an important role in regulating the DNA damage response, maintaining genomic stability and defending against aging. However, the roles of SIRT1 and SIRT6 in circadian function related to DNA damage response and to SIPS and SASP in the lung need to be clarified.

### The Role of SIRT3 in Diabetic Lung

In the STZ-induced diabetic rat model, decreased NADH/NAD^+^ redox imbalance, mitochondrial abnormalities, and decreased SIRT3 expression were present in the diabetic lung (184). Lungs from idiopathic pulmonary fibrosis patients show decreased SIRT3 activity, as indicated by acetylated mitochondrial SOD (MnSOD) levels, particularly in the lung epithelium. *Sirt3* deletion promotes lung fibrosis by augmenting mitochondrial DNA (mtDNA) damage and apoptosis in mouse alveolar epithelial cells and myofibroblasts (185, 186). SIRT3 can prevent the fibrosis phenotype via inhibition of the TGFβ1/Smad3 signaling pathway (187, 188).

#### The Role of SIRT3 in Circadian Mitochondrial Functions

Mitochondria are the factory for metabolism and energy generation in the body. SIRT3 is localized in the mitochondria and plays important roles in regulating metabolism and ROS production, maintaining mtDNA integrity and preventing aging. Analysis of the liver acetylome from the *Clock*-deficient mouse revealed that a large number of mitochondrial proteins influenced by circadian acetylation are involved in amino acid and fatty acid metabolism, glycolysis and gluconeogenesis, and the citric acid cycle (189). The core clock regulates circadian oscillation of SIRT3 activity together with oxidative enzyme activity by driving changes in NAD^+^ levels. In ClockΔ19 mice, the rhythms of SIRT3 activity are disrupted in young mice (190). MEFs and livers from *Bmal1*-deficient mice show impaired mitochondrial function due to decreased fatty acid oxidation (FAO), glucose oxidation and NAD^+^ concentrations, whereas *Cry1*- and *Cry2*-deficient mice show the opposite trend (140). The low NAD^+^ concentrations in the livers of *Bmal1* KO mice are correlated with impaired SIRT3 activity, resulting in enhanced protein acetylation and decreased enzymatic activity of SIRT3 targets, including OTC, MCAD, LCAD, MnSOD, and IDH2 (140). NAD^+^ supplementation with nicotinamide mononucleotide (NAN) restores SIRT3 activity and thereby mitochondrial oxidative capacity (140). A later study showed that both circadian and feeding rhythms coordinated the liver acetylome, including mitochondrial protein rhythmic acetylation, by impacting NAD^+^-dependent SIRT3 deacetylase activity (191). SIRT3 expression decreases in aged rats (192). As ROS are the main cause of aging and increased SIRT3 expression has been considered to contribute to human longevity, the circadian clock may influence cellular senescence and organic aging through SIRT3. In addition, SIRT3 interacts with Ku70 and deacetylates it, thus protecting cardiomyocytes from aging and stress-induced death (193). As mentioned above, the circadian clock protein also interacts with Ku70; hence, SIRT3 may be involved in circadian oscillator-mediated DNA damage/repair responses. Further studies on the circadian role of SIRT3 in the lung are needed.

### SIRT6 and Partitioning Circadian Transcription

SIRT6 is uniquely located in the nucleus and constitutively binds to the chromatin (183, 194). The genome-wide occupancy of SIRT6 is mainly at TSSs of active genomic loci, which are also binding sites for serine 5 phosphorylated RNA polymerase II (195). The chromatin binding of SIRT6 is reported to be dynamic in response to stimuli (196, 197). SIRT6 deacetylates H3K9 and H3K56 in a NAD^+^-dependent manner, regulating gene expression, genome stability and telomere maintenance, thereby impacting metabolic diseases, heart disease and cancer (198, 199). DNA microarray analysis of liver-specific *Sirt1* KO mice and liver-specific *Sirt6* KO mice revealed that SIRT6 significantly regulates hepatic CCG expression, which is exclusive to CCGs regulated by SIRT1 (59). SIRT6 interacts with the BMAL1/CLOCK complex and is responsible for chromatin recruitment of the BMAL1/CLOCK complex to the promoter regions of CCGs (59). Furthermore, SIRT6 governs circadian SREBP-1 chromatin recruitment, leading to circadian regulation of genes such as *Fasn* that are implicated in fatty acid and lipid metabolism (59). SIRT6 inhibits pulmonary fibrosis by inactivating the TGFβ1/Smad3 signaling pathway (200). Lung-targeted *Sirt6* delivery via injection of adeno-associated virus-*Sirt6* attenuates bleomycin-induced pulmonary fibrosis (200). Moreover, SIRT6 can inhibit human bronchial cell (HBEC) senescence by inactivating the TGFβ1/Smad3 signaling pathway (201). SIRT6 induces apoptosis of HBECs by attenuating the IGF-Akt-mTOR signaling pathway, which contributes to the prevention of CSE-induced HBEC senescence (202). As CSE-induced HBEC senescence has been implicated in the pathogenesis of COPD, SIRT6 is supposed to be a protective factor in chronic airway diseases (202). Consistent with this hypothesis, SIRT6 levels are positively correlated with FEV1/FVC, and its expression in the lungs of COPD patients is decreased (202). SIRT6 is speculated to be a protective factor in the diabetic lung.

Of the seven members of the Sirtuin family, only SIRT1, SIRT3, and SIRT6 have been implicated directly in circadian clock regulation, but their roles in pulmonary pathophysiology and the interactions of these Sirtuins with circadian clock are largely unknown. The majority of Sirtuins are implicated in metabolic regulation, oxidative stress, inflammation, DNA damage/repair response, and telomere length regulation, which are mainly related to aging processes as well as lung disease. Circadian rhythms are intimately related to pulmonary pathophysiology. There should be an interaction of Sirtuins and the circadian clock in lung disease, especially in metabolism-related lung disease.

## Conclusions and Future Directions

In summary, we gave a mechanistic perspective about the role of circadian clock and Sirtuins in diabetic lung based on the strengths of their roles in metabolic disturbance, oxidative stress, inflammation, and cellular DNA damage/repair responses. However, our understanding of the diabetic lung is still poor. Further studies are still needed to elucidate the following the questions. (1) Although we have summarized the potential interactions of the circadian clock and Sirtuins, the exact roles of these two systems underlying diabetic lung remain unknown. Clock genes or Sirtuins tissue-specific knock out or transgenic animals need to be used to evaluate the definite role of these molecules in diabetic lung. (2) With the development of high-throughput and epigenetic methodologies, more clearly molecular regulatory network will be identified. Therefore, more cell omic sequencing methods, which include single cell sequencing (scRNA-seq) can be used to analysis expression differences in different types of pulmonary cells and further elucidate roles of these cells in the development of diabetic lung. Moreover, assay for transposase-accessible chromatin with high-throughput sequencing (ATAC-seq) can be used to analyze epigenetic mechanisms of clock genes or Sirtuins. (3) Given the apparent roles of the circadian clock and Sirtuins in regulating a series of pathophysiologic processes and the subsequent demonstration of therapeutic value in animal models, the utility of natural or synthetic small molecules that can activate or inhibit one or more clock genes and Sirtuins would grow increasingly broader. Sirtuin-activating compounds (STACs), such as resveratrol, SRT1720 and SRT2183 (203), and synthetic REV-ERB and ROR ligands, such as GSK4112, SR9009, and SR9011 (204), have been well-documented *in vivo*. These small molecules provide potential treatment strategies for diabetic lung.

## Author Contributions

SZ and H-ZC concepted the review. SZ is responsible for literature collection and article draft. Y-MD designed the figures. X-FZ and H-ZC revised the manuscript. All authors listed have made a substantial, direct and intellectual contribution to the work, and approved it for publication.

### Conflict of Interest

The authors declare that the research was conducted in the absence of any commercial or financial relationships that could be construed as a potential conflict of interest.
